# Avian Use of Perennial Biomass Feedstocks as Post-Breeding and Migratory Stopover Habitat

**DOI:** 10.1371/journal.pone.0016941

**Published:** 2011-03-03

**Authors:** Bruce A. Robertson, Patrick J. Doran, Elizabeth R. Loomis, J. Roy Robertson, Douglas W. Schemske

**Affiliations:** 1 Kellogg Biological Station, U.S. Department of Energy Great Lakes Bioenergy Research Center, Michigan State University, East Lansing, Michigan, United States of America; 2 The Nature Conservancy, Lansing, Michigan, United States of America; 3 Battle Creek, Michigan, United States of America; 4 Department of Plant Biology, U.S. Department of Energy Great Lakes Bioenergy Research Center, Michigan State University, East Lansing, Michigan, United States of America; Institut Pluridisciplinaire Hubert Curien, France

## Abstract

Increased production of biomass crops in North America will require new agricultural land, intensify the cultivation of land already under production and introduce new types of biomass crops. Assessing the potential biodiversity impacts of novel agricultural systems is fundamental to the maintenance of biodiversity in agricultural landscapes, yet the consequences of expanded biomass production remain unclear. We evaluate the ability of two candidate second generation biomass feedstocks (switchgrass, *Panicum virgatum*, and mixed-grass prairie) not currently managed as crops to act as post-breeding and fall migratory stopover habitat for birds. In total, we detected 41 bird species, including grassland specialists and species of state and national conservation concern (e.g. Henslow's Sparrow, *Ammodramus henslowii*). Avian species richness was generally comparable in switchgrass and prairie and increased with patch size in both patch types. Grassland specialists were less abundant and less likely to occur in patches within highly forested landscapes and were more common and likely to occur in larger patches, indicating that this group is also area-sensitive outside of the breeding season. Variation in the biomass and richness of arthropod food within patches was generally unrelated to richness and abundance metrics. Total bird abundance and that of grassland specialists was higher in patches with greater vegetation structural heterogeneity. Collectively, we find that perennial biomass feedstocks have potential to provide post-breeding and migratory stopover habitat for birds, but that the placement and management of crops will be critical factors in determining their suitability for species of conservation concern. Industrialization of cellulosic bioenergy production that results in reduced crop structural heterogeneity is likely to dramatically reduce the suitability of perennial biomass crops for birds.

## Introduction

In North America, land-use changes associated with the expansion of contemporary bioenergy crops are generally expected to reduce biodiversity in affected regions [1], [2]. However, because biomass production systems (including crop selection, production and management strategies, feedstock storage and delivery) may profoundly differ in their ability to support native biodiversity, the selection of biomass crops is critical to predicting the ecological consequences of the new biofuel economy. For example, increased corn-ethanol production is likely to lead to further biodiversity losses [2], [3], but preference for next-generation perennial biomass crops such as switchgrass (*Panicum virgatum*) or mixed-grass prairie [4] may actually provide vast new acreage of available habitat for animals that require perennial grassland to survive and reproduce [2].

Perennial feedstocks can attract a number grassland bird species during the breeding season [5], [6], [7], but could also represent demographically important habitats during the non-breeding season. This potential is of particular concern for two reasons. First, grassland birds have experienced more dramatic and rapid population declines than any other group in North America [8] and represent an important component of native biodiversity likely to be impacted by the expansion of bioenergy crops. Second, much research has focused on factors shaping the stability of breeding and wintering grassland bird populations [9], but the post-breeding and migratory habitat requirements of this imperiled avifauna remains almost unstudied. This, despite the importance of these habitats to survival [10], [11]. Consequently, at a time when bioenergy crops are potentially transforming agricultural landscapes, their ability to provide important stopover and post-breeding habitat may play a significant role in the conservation of grassland bird populations.

Our goal is to directly address this information gap by comparing the bird communities in two important candidate biomass feedstocks with potential to provide post-breeding and migratory stopover habitat: Switchgrass and mixed-grass prairie. We first ask if feedstocks differ in the species richness, species density (species richness per unit area) and abundance of migratory bird communities they support, and then investigate how food availability and habitat structure and composition at multiple spatial scales (microhabitat, patch, and landscape) shape the distributions of birds during the fall migratory period. Because the post-breeding and en-route habitat requirements of grassland birds are poorly-known, we base our predictions about grassland bird responses to crops on established bird-habitat relationships during the breeding and wintering periods. Grassland bird diversity during the breeding season has been linked to plant species diversity [12] and grassland birds exhibit well-understood species-specific preferences for habitat structure [13]. Consequently, we predict that mixed prairie should support a greater diversity and abundance of migrant birds than switchgrass monoculture. We also investigate the following factors known to shape distributions of migratory birds in other systems: Food availability [14], habitat complexity [15], patch size [16] and the structure and composition of the surrounding landscape [17].

## Results

### Bird community composition

We identified 95.1% of the 979 individuals detected within transects to the species level. In total, we detected 41 bird species, with greater total and obligate species richness in mixed-grass prairie (total = 38; obligate = 8) than switchgrass (total = 30; obligate = 7, Table 1). Several species of high state and national conservation status (e.g. Grasshopper Sparrow, LeConte's Sparrow and Northern Harrier), occurred in both switchgrass and mixed prairie. No detectable, consistent year-to-year difference in community wide species richness (t = 0.48, df = 14, *P* = 0.69), species density (t = 0.18, df = 14, *P* = 0.57) or abundance (t = 0.14, df = 14, *P* = 0.55) was evident across study plots.

**Table 1:** Bird species (N = 41) detected in 15 prairie and 15 switchgrass patches in southern Michigan.

Common Name	Prairie	Switchgrass
American Crow (*Corvus brachyrhynchos*)	X	
American Goldfinch (*Spinus tristus*)	X	X
American Robin (*Turdus migratorius*)		X
American Tree Sparrow (*Spizella arborea*)	X	
Ammodramus sparrow spp.*	X	
Barn Swallow (*Hirundo rustica*)	X	X
Black-capped Chickadee (*Poecile atricapillus*)	X	
Bobolink (*Dolichonyx oryzivorus*)*	X	X
Clay-colored Sparrow (*Spizella pallida*)	X	X
Chipping Sparrow (S*pizella passerina*)	X	X
Chimney Swift (*Chaetura pelagica*)	X	
Cooper's Hawk (*Accipiter cooperii*)^1^	X	X
Common Snipe (*Gallinago gallinago*)	X	
Common Yellowthroat (*Geothlypis trichas*)	X	X
Dark-eyed Junco (*Junco hyemalis*)	X	
Eastern Bluebird (*Sialia sialis*)	X	
Eastern Meadowlark (*Sturnella magna*)*	X	X
Field Sparrow (*Spizella pusilla*)	X	
Grasshopper Sparrow (*Ammodramus savannarum*)^1^ *	X	X
House Wren (*Troglodytes aedon*)	X	X
Indigo Bunting (*Passerina cyanea*)	X	X
LeConte's Sparrow *(Ammodramus leconteii*)*	X	X
Lincoln's Sparrow (*Melospiza lincolnii*)	X	X
Northern Harrier (*Circus cyanus*)^1^ *	X	X
Nelson's Sparrow (*Ammodramus nelsoni*)^2^		X
Palm Warbler (*Dendroica palmarum*)	X	X
Ring-necked Pheasant (*Phasianus cholchicus*)	X	X
Red-tailed Hawk (*Buteo jamaicensis*)	X	X
Red-winged Blackbird (*Agalaius phoeniceus*)	X	X
Ruffed Grouse (*Bonasa umbellus*)	X	
Savannah Sparrow (*Passerculus sandwichensis*)*	X	X
Sedge Wren (*Cistothorus platensis*)*	X	X
Song Sparrow (*Melospiza melodia*)	X	X
Spizella sparrow spp.	X	X
Sharp-shinned Hawk (*Accipiter striatus*)	X	
Swamp Sparrow (*Melospiza Georgiana*)	X	X
Tennessee Warbler (*Vermivora peregrina*)	X	
Tree Swallow (*Tachycineta bicolor*)	X	X
Turkey Vulture (*Cathartes aura*)	X	X
Vesper Sparrow (*Pooecetes gramineus*)*	X	
White-crowned Sparrow (*Zonotrichia leucophrys*)	X	X
White-throated Sparrow (*Zonotrichia albicollis*)		X
Yellow-rumped Warbler (*Dendroica coronata*)	X	X
	38 (8)	30 (7)

*Obligate grassland species, Michigan species of conservation concern^1^, Audubon Watchlist species^2^ [18].

Species totals in parentheses represent obligate grassland species richness totals for prairie (n = 8) and switchgrass (n = 7).

### Arthropod communities

We captured 9,545 individual arthropods from 101 families. Mean arthropod biomass and richness were greater in mixed prairie than switchgrass (Table 2).

**Table 2:** Summary descriptions of explanatory variables from mixed-prairie (n = 15) and switchgrass patches (n = 15) in southern Michigan.

Variable		Switchgrass	Prairie	t_28_	*P*
Within-patch					
MHET	Microhabitat heterogeneity index (0–2)	0.27 (0.25)	0.43 (0.13)	3.18	0.004
MPC1	Microhabitat principal component 1	0.26 (0.55)	−0.24 (0.62)	1.40	0.18
AMAS	Arthropod biomass (g / sample)	0.006 (0.014)	0.015 (0.012)	3.10	0.004
ARIC	Arthropod richness (# families / sample)	21.99 (22.65)	43.7 (21.84)	3.31	0.002
Patch and landscape-scale					
PSIZ	Patch size (ha)	6.42 (6.38)	15.80 (13.45)	2.49	0.02
LPC1_500	Landscape principal component 1 (500m)	0.21 (0.21)	−0.2 (0.96)	1.00	0.32
LPC2_500	Landscape principal component 2 (500m)	−0.78 (1.36)	0.73 (0.49)	0.40	0.69
LDIV_500	Land cover diversity (500m) (0–1)	0.55 (0.17)	0.61 (0.22)	0.83	0.41
LPC1_1500	Landscape principal component 1 (1500m)	−0.05 (0.85)	0.05 (1.14)	0.20	0.84
LPC2_1500	Landscape principal component 2 (1500m)	−0.01 (1.23)	0.01 (0.76)	0.05	0.95
LDIV_1500	Land cover diversity (1500m) (0–1)	0.66 (0.66)	0.62 (0.09)	0.84	0.41

Means are given with standard deviations in parentheses. Critical and significance values of t-tests comparing mean values among habitats are given with *P*-values.

### Vegetation structure

Microhabitat principal component 1 was higher in switchgrass patches than prairie, indicating a higher proportion of grass vs. forbs and a higher density of vegetation (Table 2). Microhabitat heterogeneity was also slightly lower in switchgrass patches. The forb cover index in switchgrass plots (mean = 1.3, SD = 0.61) was less than that of mixed prairie (mean = 2.1, SD = 0.56). The index exceeds 1.0 in switchgrass plots because some of these patches have been invaded to some degree. Switchgrass plots were generally smaller than prairie plots, but the range of values for landscape-scale habitat variables were generally comparable between crop types (Table 2).

### Avian community metrics

**Figure 1:**
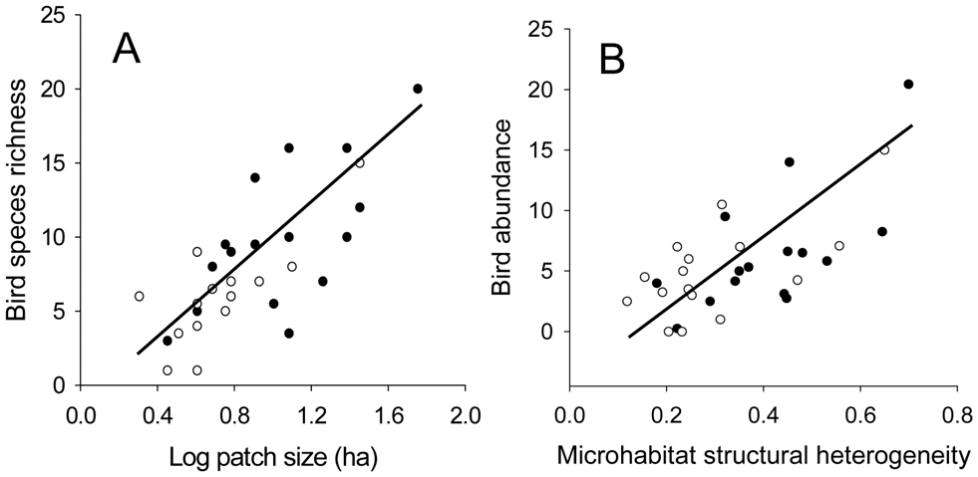
Partial regressions of (log) patch size of biomass crop patches vs. community wide species richness within a patch (A), and microhabitat structural heterogeneity vs. total bird abundance (B). Parameter estimates are based on model-averaged values. Top models did not indicate a difference in species richness or abundance between switchgrass (n = 15, open circles) and prairie habitats (n = 15, filled circles).

Global models provided a reasonable fit to the data and several global generalized linear models exhibited moderate levels of overdispersion (Table S1). Competing models of community-wide species richness indicated a positive relationship with patch size (Table 3, Figure 1a). One indicated a positive, but non-significant, relationship between community-wide bird species richness and arthropod richness, but the variable was non-significant. This was the only top model of any metric of the avian community to indicate a relationship with arthropod richness or biomass. Top models of species density indicated that density was higher in patches with greater microhabitat structural heterogeneity (MHET) within landscapes characterized by increased urbanization. Species density was also associated with reduced land cover of open and semi-natural habitats (higher values of LPC2_500), but had a non-significant p-value (Table 3).

**Table 3:** Models of avian richness, species density, abundance, and occurrence for southern Michigan bird communities in switchgrass (n = 15) and prairie patches (n = 15).

Competing models	*K* †	ΔAIC_C_ or ΔQAIC_C_	*w*_i_ ‡
Species richness (community-wide)			
1.14+0.85(PSIZ)***	2	0	0.37
1.14+0.85(PSIZ)***+6.88(AMAS)^ns^	3	0.45	0.3
Species density (community-wide)			
0.28+1.80(MHET)*	2	0	0.06
0.28+1.80(MHET)*+0.12(LPC2_500)^ns^	3	0.76	0.04
Abundance (community-wide)			
−0.16+3.22(MHET)***+0.26(LPC2_500)**	3	0	0.5
−0.16+3.22(MHET)***+1.41(LDIV_1500)*	3	1.65	0.22
Abundance (obligate species)			
−1.35+5.30(MHET)***	2	0	0.22
−1.35+5.30(MHET)***−0.2(LPC1_1500)*	3	0.61	0.16
Occurrence (obligate species)			
−5.30+4.90(PSIZ)**	2	0	0.16
−5.30+4.90(PSIZ)**+12.42(MHET)**−1.36(LPC1_1500)*	4	0.04	0.16
−5.30+12.42(MHET)**−1.36(LPC1_1500)**	3	0.15	0.15

† Number of parameters,

‡ Model Akaike weight,

*P<0.05≥0.01,

**P<0.01≥0.001,

***P<.001,

ns *P*>0.05.

The table lists the best models (ΔQAIC_c_ or ΔAIC_c_<2.00) for 1) the entire bird community, 2), breeding birds only, and 3) obligate grassland species. P-values associated with model parameters are given. Response variables: AMAS = arthropod biomass; ARIC: arthropod family richness; MPC1: microhabitat principal component 1; MHET: microhabitat heterogeneity index; CROP: biomass crop; PSIZ: log patch size; LPC1/LPC2: landscape principal components; LDIV: landscape diversity index. Landscape composition and LDIV variables are labeled with the relevant spatial scale (radius in meters from center of each patch) at which they are computed.

Microhabitat heterogeneity was important in explaining total (Figure 1b) and obligate species abundance, appearing in all top models. Landscape-scale variables linked to abundance metrics differed for each subset of the avian community (Table 3). Community-wide abundance was positively related to LPC2_500 and land cover diversity at the 1500m-radius scale in another. The abundance of grassland obligates was positively related to patch size (one model) and negatively related to LPC1_1500, indicating higher abundance in less-forested landscapes at that scale. With the exception of patch size, top models of obligate species occurrence emphasized similar variables to those explaining obligate abundance: MHET, and LPC1_1500. In an ancillary analysis, we found that model selection of obligate species occurrence using the Bayesian Information Criterion produced qualitatively similar results, emphasizing the same important independent variables as AIC_c_.

## Discussion

To our knowledge, this work represents one of the first studies to empirically assess the relative biodiversity value of candidate bioenergy feedstocks (but see [3], [7]) and to investigate the habitat requirements of grassland birds along their migratory routes (but see [19]). Few studies of post-breeding and stopover site selection have simultaneously compared the influence of factors operating at different spatial scales in explaining habitat use patterns of migratory land birds (see also [17], [20]). Most have focused efforts at a single small spatial scale [17] and research has taken place almost exclusively within forested ecosystems. We focused on both the entire bird community and on grassland specialists to best understand how factors intrinsic and extrinsic to bioenergy feedstocks may more broadly affect the ability of agricultural landscapes to act as important sources of migratory stopover and post-breeding habitat. Our results support the contention that the ability of alternative biomass feedstocks to support fall bird communities is linked to habitat characteristics at several spatial scales and that habitat characteristics favoring the settlement of specialist species differed from those favoring species richness, per se.

### Within-patch factors

Switchgrass patches were structurally dense and uniform (less heterogeneous) and had a lower forb composition than prairie reconstructions (Table 2). These attributes generally reflect the relative differences expected between agricultural monocultures and polycultures, despite the fact that the switchgrass patches we studied were not always strict monocultures. The most important within-patch factor explaining variation in avian richness, species density and abundance in both switchgrass and prairie patches was microhabitat heterogeneity (MHET, Table 3, Figure 1b). Avian richness and abundance during the fall migratory period commonly exhibits a strong positive relationship with habitat structural complexity in forested habitats [15], [16], [21], [22], [23], though some studies have reported only weak relationships [17], [24]. This inconsistent relationship might result from the cumulative effects of variable species-specific responses to habitat structure [25]. Alternatively, it could reflect the region- or habitat-specific dependence of habitat structure in mediating 1) availability of or accessibility to food [26] or 2) resource competition within or between species [27], or 3) risk of predation [28]. Because grassland birds are adapted to exploit particular ranges of structural habitat conditions [e.g. 13] and space use [29], more heterogeneous patches are more likely to provide habitat for species with different structural preferences. Predator avoidance behavior is an important determinant of grassland bird species' response to variation in microhabitat structure, affecting the tendency of certain species to flock [30]. For both of these reasons, highly heterogeneous patches are likely to provide habitat for more species. Regardless of the ultimate explanation, the pattern alone implies that more structurally diverse biomass patches will support denser, more abundant migrant communities with more grassland specialists. Richness and abundance-based metrics were related to patch-scale heterogeneity in habitat structure but not to the average vegetations characteristics of plots. This suggests that, in contrast to the breeding season, large structurally uniform areas are not required to attract species with preference for a particular microhabitat structure.

We detected several species of state and continental conservation concern in both feedstock types (Table 1). Raw species richness in switchgrass was slightly lower than that of mixed prairie, yet model selection indicated that feedstock type was a poor predictor of all avian measured community metrics we measured. Feedstock selection may still be important in shaping vegetation structural attributes that are more broadly and consistently linked to distributions of migrants. The eventual industrialization of perennial bioenergy crops will aim to maximize biomass production which, especially in monocultural systems, will likely result in a uniformly tall, dense crop structure. Yet, because prairie patches were generally more heterogeneous than switchgrass monocultures, even high biomass mixed-prairie patches may maintain the structural diversity necessary to support a relatively high diversity of birds.

The high energetic demands of migration predict that food availability should be an important component of habitat quality for migrants and those individuals preparing to migrate [15], [17], [21], [25]. Yet, recent experimental evidence suggests that arthropod abundance is not a proximate factor in habitat selection during migration [23]. Because birds are flexible in both their foraging behavior and the foods they select [31], [32], behavioral plasticity can allow migrants to effectively exploit unfamiliar and unpredictable habitats during migration [25]. We found that switchgrass supported a reduced diversity and richness of terrestrial arthropods relative to mixed-prairie patches, a pattern which also occurs in this system during the breeding season [3], [7], but found no evidence that the composition of migratory bird communities was linked to arthropod biomass in crop patches. Arthropods may be an important food source for subsets of the avian community that depend heavily on invertebrate availability, but other food resources are likely to influence migrant distributions (e.g. seed, fruit).

### Patch size

The richness of migratory bird communities increases with patch size in forested systems [16], [33]. We found this pattern to hold for migrant communities exploiting both switchgrass patches and prairies. It is unclear whether the observed richness-area effect during migration is a result of migrating individuals being more likely to intercept large patches (a.k.a ‘the target effect’) [34], or some unmeasured fitness benefit associated with larger habitat patches (e.g. reduced predation risk).

Many grassland specialist birds are ‘area-sensitive’ (i.e. more likely to occur in large habitat patches than smaller ones) during the breeding season [35], which can manifest as a positive relationship between species density and patch size [36]. As expected, community-wide species density did not increase with patch size during the migratory period, yet obligate species abundance and occurrence were positively linked to patch size. This is consistent with the findings of [33] who demonstrated that forest-dwelling species that were area sensitive during the breeding season were also area sensitive during the migratory period. Lack of area-sensitivity at the community level in this study is not surprising because many species are not restricted to grassland patches.

### Landscape structure and composition

Grassland specialist birds commonly avoid selecting breeding habitat within highly forested landscapes [37], [38], [39], which is a mechanism shaping area-sensitivity in this species group [35]. We observed forest avoidance (1500m-radius scale) during migration for obligate grassland species (Table 3). That species density and abundance were not linked to forest cover during migration is expected given that the overall species pool contains many species unrestricted to grassland habitats.

Instead, top models of species density and abundance were positively linked with decreasing cover of low, open habitat types and increasing urban land cover. Other studies linking migrant diversity and abundance to surrounding land cover composition are lacking, but this result is surprising given that species richness of breeding bird species decline in relation to increasing urbanization at local and landscape scales [40]. Attraction to urban areas could be explained by enhanced foraging opportunities (e.g. bird feeders), reduced predation risk or flexibility in habitat use by migrants in exploiting habitats not previously considered to be suitable [41]. Recent research demonstrates that even small patches of habitat in urban landscapes provide adequate food and protection for some species of migrants that are positively area-sensitive during the breeding season [42], [43]. Alternatively migrants could be concentrating where few other suitable habitats exist (e.g. more urbanized areas). Species density was also positively associated with landscape diversity at the 1.5 km scale, suggesting that habitat diversity within landscapes enhances the local diversity of migratory birds within focal habitats. Collectively, these results imply that cultivation of perennial-based biomass feedstocks in less-forested landscapes will be required to enhance habitat for grassland specialists, but that switchgrass and prairie reconstructions can provide post-breeding or migratory stopover habitat for a broad diversity of bird species even when they occur within urbanized or forested landscapes.

### Conclusions

Assessing the biodiversity impact of novel production systems is fundamental to reconciling the demands of biodiversity conservation and agricultural production [44]. Loss of native grassland ecosystems throughout most of North America has exceeded 90% [45], and agricultural grassland habitats have become critical to the maintenance of populations of many grassland bird species [46], [47]. Our results suggest that candidate perennial biofuel feedstocks have potential to provide a source of post-breeding and migratory habitat to avifauna of high conservation importance. The value of these habitats may be especially high where they replace contemporary biomass crops (e.g. corn) [2], [7]. While we draw inferences from bird-habitat relationships based on extant variation in within-patch habitat structure, most of the patches we studied were not actively managed for biomass production. Ultimately, biomass production systems could include chemical inputs (e.g. fertilizers, herbicides) and the selection of high-biomass genotypes that could reduce plant species diversity [48] and structural heterogeneity, especially in monocultural systems.

Results of this study suggest that the latter effect is likely to dramatically reduce the suitability of biomass crops as autumn stopover habitat. Switchgrass and prairie are generally expected to be harvested in September, but are somewhat flexible. Harvest strategies that create within-crop structural diversity (e.g. strip harvesting) or that produce a mosaic of harvested and unharvested patches during the migratory period could be a useful management tool [1], [5], [49]. Because crop management schemes may profoundly affect the biodiversity and ecosystem services value of biomass crops, empirical research is needed to understand how harvest strategies and schedules and chemical inputs can alter the value of crops to birds and other taxa. The value of stopover habitat to migrants is commonly linked to the efficiency of refueling which, in turn, can potentially affect migration timing and success [50]. Because we found that arthropod biomass differed between feedstocks, an explicit evaluation of the refueling value of perennial monocultures vs. polycultures is needed. North American countries have not yet adopted bioenergy-related standards to protect biodiversity, but the development of such policies should draw on lessons from a parallel situation in Europe where biofuel expansion and reductions in targets for set-aside programs have already had negative impacts on grassland bird populations [51].

## Materials and Methods

### Study design and site selection

**Figure 2:**
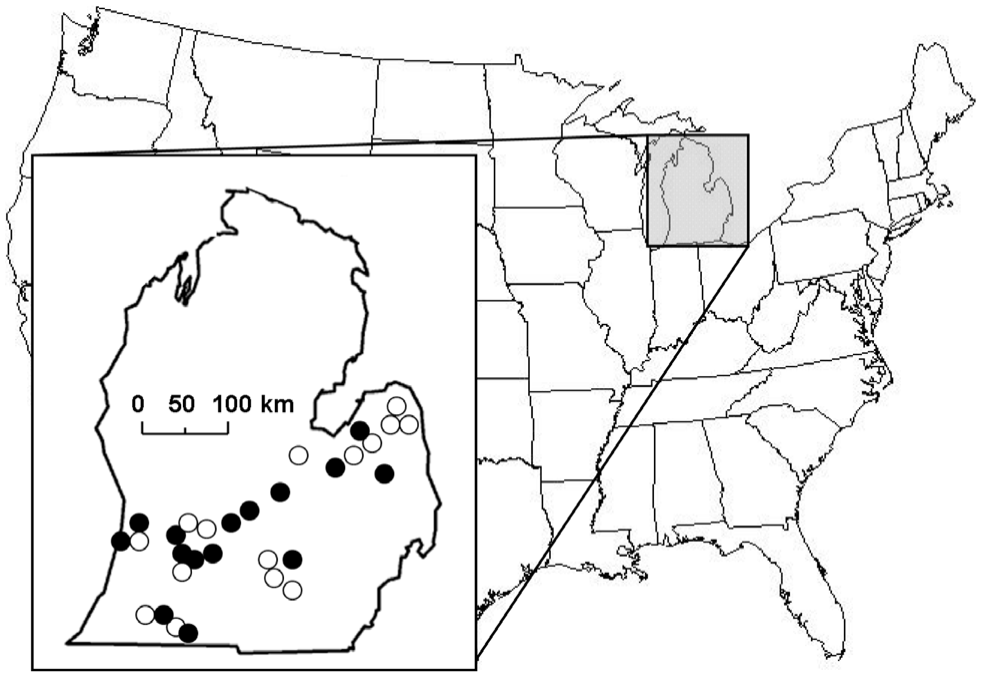
Map of the study region in the southern peninsula of Michigan. Locations of mixed-grass prairie (n = 15, filled circles) and switchgrass (n = 15, open circles) study sites are indicated.

Fifteen sites of each of the two biomass crop treatments were selected from established patches throughout southern Michigan (Figure 2). We visited 19 sites in 2008 (prairie = 12, switch = 7) and 26 sites in 2009 (prairie = 11, switch = 15), and surveyed a subset of plots in both years (prairie = 8, switchgrass = 7) to examine inter-annual variation in the avian community. Market demand for perennial biomass crops is still extremely low, improvement (e.g. maximizing biomass, minimizing nutrient demand) of genotypes for perennial biomass candidates (e.g. switchgrass) is ongoing, and research investigating optimal species mixtures for polycultural crops has not yet occurred, making it impossible to study the impact of perennial biomass crops intensively managed for biomass production. Grassland birds specialize in grasslands differing in their physical structure [12] and bioenergy production systems should focus on feedstocks and management techniques that maximize biomass. Consequently, we chose to investigate vegetation structural attributes we feel are most likely to be affected by feedstock selection and management and relevant to bird community composition: 1) vegetation height and density and 2) variation in vegetation structure. We first identified all known patches planted in switchgrass and mixed-grass prairie throughout the southern half of the southern peninsula of the state of Michigan. Because most patches were not actively managed for biomass production, but primarily for wildlife habitat or as native community restorations, switchgrass patches were not always strict monocultures. Within each treatment, we selected sites representing a range of crop height and stand structural heterogeneity from within landscapes varying as much as possible in the amount of non-crop habitat they contained. Because we wished to examine the importance of patch size in shaping avian communities, we also selected patches to vary as widely as possible in size (Prairie: 2–55ha; Switchgrass: 2–32.3ha). Study patches were located a minimum distance of 5 km from other sites.

### Bird Surveys

We surveyed the bird community associated with crop patches in the fall of 2008 and 2009, making three visits to each patch: 1) Sep. 7–Sep. 15, 2) Sep. 23–Oct 1, and 3) Oct 7–16. While this time period corresponds to the period of peak migratory movement for migrant land birds in this region [52], birds in study patches also include those dispersing post-fledging and staging prior to migration as well as a small number of resident species whose over-winter abundance is significantly reduced (e.g. *Melospiza melodia*) [52]. No grassland specialist species over-winter in this region. Species richness and abundance were estimated based on two survey techniques: strip transects and area searches. We chose these two techniques because area-search techniques produce more accurate species richness estimates while rope-drag-type strip transects (described below) produce more accurate estimates of abundance for non-breeding grassland birds [53]. No relevant distance sampling technique has yet been developed to improve the accuracy of these techniques.

Fixed-width transects [54] were 100m long by 25m wide. Because grassland birds are difficult to detect when not singing we employed a rope drag technique in which a 25m-long rope connecting two observers was dragged across vegetation to flush birds, increasing their visual detectability and the likelihood of birds to produce species-specific vocalizations that could be used in identification. Flushed birds were identified visually when perched or in flight and by species-specific call notes and were tracked until they landed to ensure to avoid double-counting individual in the same or other transects. This technique was specifically designed to increase the detectability of grassland birds during the non-breeding season and has been shown to increase observer efficiency and bird detectability over fixed-distance point counts [55]. Compared to traditional strip-transect surveys, this technique employs twice as many observers, covers a relatively narrow strip of habitat (25 m vs. ≥50 m) and allows observers to focus attention on a more narrow angle of vision (90° vs. 180°). Consequently, our technique should also provide substantial improvements in detectability over typical strip-transects [55]. Individuals that could not be assigned to species were recorded as “unknown” or identified to the genus or family level (e.g. *Ammodramus* spp.). Observations of individuals not identified to the species level were used only to estimate community-wide abundance. Bird surveys were conducted during the first four hours after sunrise.

In order to obtain representative samples of bird communities in patches differing in area without pseudo-replicating [37] we varied the number of transects sampled per patch, then aggregated information for each patch prior to analysis. The smallest patches contained a single transect while the number of transects surveyed per patch increased with patch size up to six in the largest patches. Transects were oriented and surveyed in a linear series such that no transect began or ended closer than 50m from the edge of each patch and one transect ran through the geographic center of the patch. Grassland bird communities exhibit ‘area-sensitivity’, or increased species density (species richness per unit area) in larger habitat patches [36]. We calculated patch-scale species density as the median value of species richness within each transect in a patch, combining data from both site visits. We calculated patch-scale abundance as the median value of total bird abundance within each patch, combining data from both site visits. We used species density as a metric to test the hypothesis that avian communities are also area-sensitive during the post-breeding and migratory period in both prairie and switchgrass crops.

To estimate patch-scale species richness, we used area searches to survey portions of each patch not covered by transects. To maintain observer effort proportional to the size of each patch, observers walked at a regular pace though each patch in a systematic pattern such that one observer passed within 75 m of every point in a patch exactly once. Species detected during strip-transect surveys, including those detected at a distance of >50 m, were pooled with detections from area searches to provide an estimate of bird species richness within each patch.

### Within-patch habitat structure

During the second site visit we characterized vegetation structure of crops within each 100 m-long transect to determine how microhabitat gradients may affect spatial distributions of birds. We randomly selected five non-overlapping sampling points within each transect at which we recorded vertical density of vegetation and canopy coverage. Vertical density (an index of biomass) [56] was quantified by measuring the minimum height of visual obstruction from 4m in each cardinal direction from a Robel pole at a height of 1m [57]. Canopy coverage was estimated on the basis of non-overlapping percentages of forbs and grass using a Daubenmire quadrat viewed from 1.5 m directly above [58]. Cover estimates were assigned an index number corresponding to a range of vegetation coverage (1 = 0–5%, 2 = 5–25%, 3 = 25–50%, 4 = 50–75%, 5 = 75–95%, 6 = 95–100%). Mean values of microhabitat variables were computed at the patch-scale. We also used variation in density within patches to calculate a patch-scale index of habitat heterogeneity originally created to capture variation in habitat structure relevant to grassland birds [59]. The index is based upon the sum of the difference between the maximum and minimum values of density taken within each transect and the sum of the mean values of the density metric for each transect:
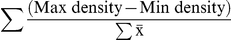



### Patch and landscape variables

Settlement behavior in grassland birds is frequently linked to landscape composition at larger spatial scales (1000–1600m) [36], [37], [38], but more local scales may be relevant during migration. We characterized landscape composition and diversity within 0.5 km and 1.5 km radii surrounding study sites using the 2009 Cropland Data Layer (56 m resolution) [60]. We categorized patches as containing cropland (e.g. corn, soybeans), herbaceous perennial habitats (including grasslands), forest, urban land (>60% impervious surface). We pooled all other land cover classes into a fifth category (<1% of total area) that were excluded from analyses. The accuracy of land-use categories was directly verified during site visits. The proportion of the landscape within 0.5 and 1.5 km of each site in these cover types was calculated using ArcGIS 9.3 [61]. We used the Patch Analyst 4.0 extension to ArcGIS to calculate a modified Simpson's Diversity Index [62].

We used principal components analysis to reduce the number of within-patch vegetation structural and landscape-scale variables at the 0.5 km and 1.5 km scales into component variables. We employed an orthogonal rotation method that minimizes the number of variables with high loadings on each axis. Microhabitat variables were moderately correlated (Table S2) and we extracted a single principal component describing microhabitat structure (MPC1) accounting for 46% of the total variation (eigenvalue 1.38, Table S3) which described a gradient of increasing grass cover and density and decreasing forb cover (Table S4). Landscape components were correlated (Table S5). The first landscape component at the 500-m-radius (LPC1_500) accounted for 50% of the total variation (eigenvalue 2.01, Table S6) and described a gradient of increasing row crops and open habitats and decreasing forest cover in the landscape (Table S7). The second landscape component at the 500-m-radius (LPC2_500) accounted for 31% of the total variation (eigenvalue 1.24) and described a gradient of increasing urbanization and reduced open and semi-natural habitats. The first landscape component at the 1500-m-radius (LPC1_1500) accounted for 46% of the total variation (eigenvalue 1.86, Tables S8, S9) and described a gradient of increasing forest and decreasing row crops in the landscape (Table S10). The second landscape component at the 1500-m-radius (LPC2_1500) accounted for 32% of the total variation (eigenvalue 1.29) and described a gradient of increasing open habitats and decreasing urbanization.

### Arthropod richness and biomass

Arthropod food availability has been linked to the distributions of post-breeding and migratory birds [14], [63]. We sampled terrestrial arthropods via sweep net samples of above-ground vegetation near the geographic center of each patch during the second site. Each of two within-patch sweep sample transects began at a distance of 50m in opposite directions from the patch center on a north-south axis. Each sample consisted of fifty sweeps taken while slowly moving toward the plot center. Both within-patch samples were combined and sealed in plastic bags and transferred to 90% ethanol solution for storage. Individuals were later identified to the family level and their length measured. We estimated individual mass using published length-regression estimates [64], [65] and then computed total arthropod biomass at the patch-level. Patch-scale estimates of arthropod family richness were obtained using the Chao 1 asymptotic richness estimators in the program EstimateS [66].

### Statistical analysis

We tested for spatial autocorrelation among sites by comparing residuals of bird community models by using the Moran's index (I) as a function of spatial distance [67] using the R package [68]. Because correlograms of Moran's I at various distance lags and the resulting correlogram [69] showed no evidence of spatial dependence among observations we did not take into account any spatial autocovariate in the models.

We took a model selection approach to determine the relative importance and effect size of 11 environmental variables and feedstock type (Table 2) in explaining variation in the richness, species density, occurrence and abundance of 1) grassland obligate bird species and 2) the entire avian community. We considered species grassland obligates based on published research demonstrating that their breeding habitat is entirely or largely restricted to natural or semi-natural grassland habitat (sensu [70], see Table 1). Because bird communities may undergo annual variation that could bias model selection, we tested for differences among years in community metrics using paired t-tests for sites that were visited in both years of the study.

We modeled the richness of the avian community using generalized linear models with a Poisson distribution and log-link function using SPSS version 15 [71]. For sites that were surveyed during two years, we took the mean value of all independent variables and median values of avian community metrics combining both years. These sites were given twice the weight in analyses. We modeled the likelihood of any obligate grassland bird occurring in a crop patch using binary logistic regression [72]. Because species richness generally increases with patch size in an asymptotic and non-linear fashion [73], we log transformed the patch size prior to analysis.

We developed a set of *a priori* candidate models that reflected our assessment of likely causes of variation in richness, species density, occurrence and abundance. Our analyses included models of each explanatory variable alone, and two- and three-variable models that we determined to be ecologically relevant. Because species-area relationships may differ by feedstock, we also included models with interactions between patch size and feedstock type. We evaluated the degree of support for logistic models using Akaike's second-order information criterion with a small sample size adjustment (AIC_c_) [74]. Because count data are commonly overdispersed, we used QAIC_c_ (quasi-AIC_c_) which accounts for potential overdispersion of generalized linear models [75]. We judged degree of support for models using ▵AIC_c_ or ▵QAIC_c_ values and normalized Akaike weights (*w_i_*). We considered models with ▵AIC_c_ or QAIC_c_ 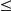
2 to have substantial support and models with ▵AIC_c_ or QAIC_c_>2 and 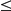
4 w to have little to no empirical support [75]. We assessed the fit of global generalized linear models (models including all factors) using c-hat [76] and the fit of the global logistic regression models with a goodness of fit test [75, Supporting Text 1]. Relationships with dependent variations are based upon model-averaged estimates [75].

## Supporting Information

Table S1 Fit of global models for avian community metrics in model selection analyses. The fit of generalized linear models is assessed as c-hat. A c-hat approximating 1 indicates good fit, while a value greater than 1, but less than 4 indicates moderate to severe overdispersion [76]. Fit of the global logistic regression model was assessed with a Hosmer and Lemeshow goodness of fit test [72] (see methods), in which the null hypothesis is that there is no difference between the observed and predicted values of the dependent variable.
(DOCX)

Table S2 Correlation matrix of microhabitat vegetation structural and composition variables. Microhabitat variables describing the structure and composition of biofuel crop stands were moderately-correlated.
(DOCX)

Table S3 Eigenvalues of the first three orthogonal microhabitat principal components extracted.
(DOCX)

Table S4 Loading matrix for the first microhabitat principal component. The principal component was positively related to vertical density and percent grass cover, and negatively related to forb cover.
(DOCX)

Table S5 Correlation matrix of land-use categories in landscapes surrounding focal patches at the 0.5 km scale. The percent cover of forest in landscapes surrounding biofuel crops was negatively correlated with crop cover, urbanization and open habitat types at the 0.5 km scale.
(DOCX)

Table S6 Eigenvalues of the first four orthogonal landscape principal components extracted at the 0.5 km scale.
(DOCX)

Table S7 Loading matrix for the first two landscape principal components at the 0.5 km scale. Landscape principal component 1 exhibited a strong positive relationship with the cover of cropland and open habitats, while landscape principal component 2 was most strongly characterized by a strong positive relationship with urbanization.
(DOCX)

Table S8 Correlation matrix of land-use categories in landscapes surrounding focal patches at the 1.5 km scale. The percent cover of forest in landscapes surrounding biofuel crops was negatively correlated with crop cover while urban cover and open habitat types were also negatively correlated at the 1.5 km scale.
(DOCX)

Table S9 Eigenvalues of the first four orthogonal landscape principal components extracted at the 1.5 km scale.
(DOCX)

Table S10 Loading matrix for the first two landscape principal components at the 1.5 km scale. Landscape principal component 1 exhibited a strong positive relationship with forest cover and a strong negative relationship with crop cover, while landscape principal component 2 exhibited a strong negative relationship with urbanization and a positive relationship with open habitats including old fields, prairie, switchgrass and pasture.
(DOCX)
