# Supplementary material for: Avian Use of Perennial Biomass Feedstocks as Post-Breeding and Migratory Stopover Habitat
Source: PLoS One. 2011 Mar 3;6(3):e16941. doi: 10.1371/journal.pone.0016941 (PMC3048387; doi:10.1371/journal.pone.0016941)
Supplement: Table S1 — Fit of global models for avian community metrics in model selection analyses. The fit of generalized linear models is assessed as c-hat. A c-hat approximating 1 indicates good fit, while a value greater than 1, but less than 4 indicates moderate to severe overdispersion [76]. Fit of the global logistic regression model was assessed with a Hosmer and Lemeshow goodness of fit test [72] (see methods), in which the null hypothesis is that there is no difference between the observed and predicted values of the dependent variable. (DOCX) [file pone.0016941.s001.docx]

Table S1

| Community metric | |  | c-hat |
| --- | --- | --- | --- |
|  |  |  |  |
| Species richness | |  |  |
|  | Entire community |  | 1.706 |
|  |  |  |  |
| Species density | |  |  |
|  | Entire community |  | 0.917 |
|  |  |  |  |
| Abundance | |  |  |
|  | Entire community |  | 2.643 |
|  | Obligate species |  | 1.838 |
|  |  |  |  |
| Occurrence | |  |  |
|  | Obligate species |  | $\chi^{2}$= 9.53 |
|  |  |  | df = 8, *P* =0.3 |
|  |  |  |  |
