# Supplementary material for: Avian Use of Perennial Biomass Feedstocks as Post-Breeding and Migratory Stopover Habitat
Source: PLoS One. 2011 Mar 3;6(3):e16941. doi: 10.1371/journal.pone.0016941 (PMC3048387; doi:10.1371/journal.pone.0016941)
Supplement: Table S2 — Correlation matrix of microhabitat vegetation structural and composition variables. Microhabitat variables describing the structure and composition of biofuel crop stands were moderately-correlated. (DOCX) [file pone.0016941.s002.docx]

Table S2.

| Variable | | % grass cover |  | % forb cover |  | Vertical density |
| --- | --- | --- | --- | --- | --- | --- |
|  |  |  |  |  |  |  |
|  | % grass cover | 1 |  | -0.24 |  | 0.22 |
|  | % forb cover | - |  | 1 |  | -0.11 |
|  | Vertical density | - |  | - |  | 1 |
|  |  |  |  |  |  |  |
