# Supplementary material for: Avian Use of Perennial Biomass Feedstocks as Post-Breeding and Migratory Stopover Habitat
Source: PLoS One. 2011 Mar 3;6(3):e16941. doi: 10.1371/journal.pone.0016941 (PMC3048387; doi:10.1371/journal.pone.0016941)
Supplement: Table S3 — Eigenvalues of the first three orthogonal microhabitat principal components extracted. (DOCX) [file pone.0016941.s003.docx]

Table S3.

|  |  |  | Initial eigenvalues | | |
| --- | --- | --- | --- | --- | --- |
|  |  |  |  |  |  |
| Component | |  | Total |  | % of variance |
|  |  |  |  |  |  |
|  | 1 |  | 1.38 |  | 46.11 |
|  | 2 |  | 0.89 |  | 29.82 |
|  | 3 |  | 0.72 |  | 24.03 |
|  |  |  |  |  |  |
