# Supplementary material for: Avian Use of Perennial Biomass Feedstocks as Post-Breeding and Migratory Stopover Habitat
Source: PLoS One. 2011 Mar 3;6(3):e16941. doi: 10.1371/journal.pone.0016941 (PMC3048387; doi:10.1371/journal.pone.0016941)
Supplement: Table S4 — Loading matrix for the first microhabitat principal component. The principal component was positively related to vertical density and percent grass cover, and negatively related to forb cover. (DOCX) [file pone.0016941.s004.docx]

Table S4.

| Variable | |  | Component 1 |
| --- | --- | --- | --- |
|  |  |  |  |
|  | Vertical density |  | 0.61 |
|  | % forb cover |  | -0.65 |
|  | % grass cover |  | 0.61 |
|  |  |  |  |
