# Supplementary material for: Avian Use of Perennial Biomass Feedstocks as Post-Breeding and Migratory Stopover Habitat
Source: PLoS One. 2011 Mar 3;6(3):e16941. doi: 10.1371/journal.pone.0016941 (PMC3048387; doi:10.1371/journal.pone.0016941)
Supplement: Table S5 — Correlation matrix of land-use categories in landscapes surrounding focal patches at the 0.5 km scale. The percent cover of forest in landscapes surrounding biofuel crops was negatively correlated with crop cover, urbanization and open habitat types at the 0.5 km scale. (DOCX) [file pone.0016941.s005.docx]

Table S5.

| Vertical density | | % crop |  | % open habitats |  | % forested |  | % urban |
| --- | --- | --- | --- | --- | --- | --- | --- | --- |
|  |  |  |  |  |  |  |  |  |
|  | % crop | 1 |  | 0.14 |  | -0.69 |  | 0.09 |
|  | % open habitats | - |  | 1 |  | -0.64 |  | -0.31 |
|  | % forested | - |  | - |  | 1 |  | -0.67 |
|  | % urban | - |  | - |  | - |  | 1 |
|  |  |  |  |  |  |  |  |  |
