# Supplementary material for: Avian Use of Perennial Biomass Feedstocks as Post-Breeding and Migratory Stopover Habitat
Source: PLoS One. 2011 Mar 3;6(3):e16941. doi: 10.1371/journal.pone.0016941 (PMC3048387; doi:10.1371/journal.pone.0016941)
Supplement: Table S6 — Eigenvalues of the first four orthogonal landscape principal components extracted at the 0.5 km scale. (DOCX) [file pone.0016941.s006.docx]

Table S6.

|  |  |  | Initial eigenvalues | | |
| --- | --- | --- | --- | --- | --- |
|  |  |  |  |  |  |
| Component | |  | Total |  | % of variance |
|  |  |  |  |  |  |
|  | 1 |  | 2.01 |  | 50.29 |
|  | 2 |  | 1.23 |  | 30.98 |
|  | 3 |  | 0.65 |  | 16.13 |
|  | 4 |  | 0.10 |  | 2.59 |
|  |  |  |  |  |  |
