# Supplementary material for: Avian Use of Perennial Biomass Feedstocks as Post-Breeding and Migratory Stopover Habitat
Source: PLoS One. 2011 Mar 3;6(3):e16941. doi: 10.1371/journal.pone.0016941 (PMC3048387; doi:10.1371/journal.pone.0016941)
Supplement: Table S7 — Loading matrix for the first two landscape principal components at the 0.5 km scale. Landscape principal component 1 exhibited a strong positive relationship with the cover of cropland and open habitats, while landscape principal component 2 was most strongly characterized by a strong positive relationship with urbanization. (DOCX) [file pone.0016941.s007.docx]

Table S7.

| Variable | |  | Component 1 |  | Component 2 |
| --- | --- | --- | --- | --- | --- |
|  |  |  |  |  |  |
|  | % crop |  | 0.74 |  | 0.44 |
|  | % open habitats |  | 0.73 |  | -0.52 |
|  | % forested |  | -0.96 |  | -0.13 |
|  | % urban |  | -0.84 |  | 0.87 |
|  |  |  |  |  |  |
