# Supplementary material for: Avian Use of Perennial Biomass Feedstocks as Post-Breeding and Migratory Stopover Habitat
Source: PLoS One. 2011 Mar 3;6(3):e16941. doi: 10.1371/journal.pone.0016941 (PMC3048387; doi:10.1371/journal.pone.0016941)
Supplement: Table S8 — Correlation matrix of land-use categories in landscapes surrounding focal patches at the 1.5 km scale. The percent cover of forest in landscapes surrounding biofuel crops was negatively correlated with crop cover while urban cover and open habitat types were also negatively correlated at the 1.5 km scale. (DOCX) [file pone.0016941.s008.docx]

Table S8.

| Vertical density | | % crop |  | % forested |  | % urban |  | % open habitats |
| --- | --- | --- | --- | --- | --- | --- | --- | --- |
|  |  |  |  |  |  |  |  |  |
|  | % crop | 1 |  | -0.83 |  | -0.22 |  | -0.89 |
|  | % forested | - |  | 1 |  | 0.08 |  | -0.16 |
|  | % urban | - |  | - |  | 1 |  | -0.31 |
|  | % open habitats | - |  | - |  | - |  | 1 |
|  |  |  |  |  |  |  |  |  |
