# Supplementary material for: Avian Use of Perennial Biomass Feedstocks as Post-Breeding and Migratory Stopover Habitat
Source: PLoS One. 2011 Mar 3;6(3):e16941. doi: 10.1371/journal.pone.0016941 (PMC3048387; doi:10.1371/journal.pone.0016941)
Supplement: Table S10 — Loading matrix for the first two landscape principal components at the 1.5 km scale. Landscape principal component 1 exhibited a strong positive relationship with forest cover and a strong negative relationship with crop cover, while landscape principal component 2 exhibited a strong negative relationship with urbanization and a positive relationship with open habitats including old fields, prairie, switchgrass and pasture. (DOCX) [file pone.0016941.s010.docx]

Table S10.

| Variable | |  | Component 1 |  | Component 2 |
| --- | --- | --- | --- | --- | --- |
|  |  |  |  |  |  |
|  | % crop |  | -0.96 |  | 0.06 |
|  | % forested |  | 0.95 |  | -0.01 |
|  | % urban |  | 0.07 |  | -0.82 |
|  | % open habitats |  | 0.01 |  | 0.80 |
|  |  |  |  |  |  |
